# Supplementary material for: EIDD-3525 is a broad-spectrum antiviral against alphavirus infection that targets the nsP4 RNA-dependent RNA polymerase
Source: bioRxiv. 2026 Sep 28:2026.09.26.754626. Preprint. [Version 1] doi: 10.64898/2026.09.26.754626 (PMC13648489; doi:10.64898/2026.09.26.754626)
Supplement: Supplementary file 1 [file NIHPP2026.09.26.754626v1-supplement-1.pdf]

bioRxiv preprint doi: https://doi.org/10.64898/2026.09.26.754626; this version posted September 28, 2026. The copyright holder for this preprint (which was not certified by peer review) is the author/funder, who has granted bioRxiv a license to display the preprint in perpetuity. It is made available under a CC-BY 4.0 International license.

Fig. S1. Tissue distribution of EIDD-3525. (**A**) Schematic of experimental design created with Biorender.com. WT C57BL/6 mice (n = 4 vehicle; n = 8 EIDD-3525) were treated by oral gavage with vehicle only or 300 mg/kg EIDD-3525. Treatment continued once per day until the pre-determined experimental endpoint at 7 days post-treatment initiation. To assess toxicity of the treatment, mouse body weights were measured daily. At 24 h after the final treatment, tissues were collected, weighed, and analyzed. (**B**) The percentage of body weight relative to initial body weight over time. Statistical significance was determined by repeated measures ANOVA with Tukey's multiple comparison test. ns not significant. (**C and D**) The amount of EIDD-3525 (**C**) and its active 5′-triphosphate metabolite, EIDD-3667 (**D**) in tissues was quantitated. Data show the mean ± SD of measurements on 8 mice.
